# ESBL plasmids in *Klebsiella pneumoniae*: diversity, transmission and contribution to infection burden in the hospital setting

**DOI:** 10.1186/s13073-022-01103-0

**Published:** 2022-08-23

**Authors:** Jane Hawkey, Kelly L. Wyres, Louise M. Judd, Taylor Harshegyi, Luke Blakeway, Ryan R. Wick, Adam W. J. Jenney, Kathryn E. Holt

**Affiliations:** 1 Department of Infectious Diseases, Central Clinical School, Monash University, Melbourne, VIC Australia; 2 Microbiology Unit & Department of Infectious Diseases, The Alfred Hospital, Melbourne, VIC Australia; 3 Department of Infection Biology, London School of Hygiene and Tropical Medicine, London, UK

**Keywords:** ESBL, Plasmids, *Klebsiella pneumonia*, Third-generation cephalosporin resistance

## Abstract

**Background:**

Resistance to third-generation cephalosporins, often mediated by extended-spectrum beta-lactamases (ESBLs), is a considerable issue in hospital-associated infections as few drugs remain for treatment. ESBL genes are often located on large plasmids that transfer horizontally between strains and species of Enterobacteriaceae and frequently confer resistance to additional drug classes. Whilst plasmid transmission is recognised to occur in the hospital setting, the frequency and impact of plasmid transmission on infection burden, compared to ESBL + strain transmission, is not well understood.

**Methods:**

We sequenced the genomes of clinical and carriage isolates of *Klebsiella pneumoniae* species complex from a year-long hospital surveillance study to investigate ESBL burden and plasmid transmission in an Australian hospital. Long-term persistence of a key transmitted ESBL + plasmid was investigated via sequencing of ceftriaxone-resistant isolates during 4 years of follow-up, beginning 3 years after the initial study.

**Results:**

We found 25 distinct ESBL plasmids. We identified one plasmid, which we called Plasmid A, that carried *bla*_CTX-M-15_ in an IncF backbone similar to pKPN-307. Plasmid A was transmitted at least four times into different *Klebsiella* species/lineages and was responsible for half of all ESBL episodes during the initial 1-year study period. Three of the Plasmid A-positive strains persisted locally 3–6 years later, and Plasmid A was detected in two additional strain backgrounds. Overall Plasmid A accounted for 21% of ESBL + infections in the follow-up period.

**Conclusions:**

Here, we systematically surveyed ESBL strain and plasmid transmission over 1 year in a single hospital network. Whilst ESBL plasmid transmission events were rare in this setting, they had a significant and sustained impact on the burden of ceftriaxone-resistant and multidrug-resistant infections. If onward transmission of Plasmid A-carrying strains could have been prevented, this may have reduced the number of opportunities for Plasmid A to transmit and create novel ESBL + strains, as well as reducing overall ESBL infection burden.

**Supplementary Information:**

The online version contains supplementary material available at 10.1186/s13073-022-01103-0.

## Background

Healthcare-associated infections (HAIs) are a top global health priority. In industrialised countries, the HAI burden is greater than that of all other communicable diseases combined [1]. Antimicrobial resistant (AMR) HAIs are particularly problematic as they reduce treatment options, and the World Health Organization recognises third-generation cephalosporin-resistant (3GCR) and carbapenem-resistant Gram-negative bacterial HAI pathogens, including *Klebsiella pneumoniae* and *Escherichia coli*, as a critical threat [2]. These resistance phenotypes are typically mediated by horizontally acquired extended-spectrum beta-lactamase (ESBL) and carbapenemase genes, encoded on large plasmids that circulate amongst different species. As a result, transmission of AMR HAIs within healthcare settings can result from transmission of a resistant strain, or of a resistance-encoding plasmid between strains of the same or different species [3]. *K. pneumoniae* is a well-known reservoir of AMR plasmids, recognised for its ability to acquire and transfer these plasmids across its population and into other species [4, 5].

Whole genome sequencing (WGS) is a powerful approach for investigating transmission of AMR HAIs, which is gradually being implemented to detect and contain transmission of AMR pathogens [6–8]. Lengthy hospital outbreaks (3–6 years) have been documented due to plasmid transmission (frequently involving *Klebsiella*) [9–11], and metagenomic studies show that AMR plasmids are ubiquitous in hospitals [12]. However, genomics surveillance studies typically focus on identifying strain transmission rather than plasmid transmission [8], and the contribution of the latter to HAI burden remains poorly understood.

We have previously reported WGS data from the year-long *Klebsiella* Acquisition Surveillance Project at Alfred Health (KASPAH), which involved the following: (i) collection of all clinical isolates identified as *K. pneumoniae* in the Alfred Hospital Microbiological Diagnostic Laboratory, which serves the Alfred Hospital (a large tertiary hospital with a large trauma and intensive care unit (ICU)) and three smaller hospitals (Caulfield, Bethlehem, Sandringham), together known as the Alfred Health network [13]; (ii) screening for rectal or throat colonisation with *K. pneumoniae* in the ICU and two geriatric wards in the Caulfield hospital [14, 15]; (iii) screening for rectal colonisation with any 3GCR Gram negative bacteria in the ICU (substudy during the final three months) [16]. The ICU substudy showed that the majority of *K. pneumoniae* infections in ICU patients (~ 80%) resulted from *K. pneumoniae* colonising the patients’ gut on admission to hospital, with < 20% of cases [14] resulting from nosocomial transmission. Analysis of the full collection of Alfred Health network clinical isolates reflected a similar pattern, estimating just 10% of *K. pneumoniae* infections resulted from nosocomial transmission [13]. Importantly ESBL carriage was the most significant risk factor for nosocomial transmission (OR 21, *p* < 1 × 10^−11^ in a logistic regression model; estimated risk of onward transmission was 28% for ESBL strains vs 1.7% for non-ESBL strains), and transmission of ESBL strains was shown to be responsible for doubling the prevalence of ESBL + *K. pneumoniae* from 15% in the first 9 months of the study to > 30% in the final 3 months [13]. The vast majority of acquired AMR genes amongst *K. pneumoniae* clinical infections was found to be plasmid-encoded, and the most common ESBL gene was *bla*_CTX-M-15_; however, plasmid transmission was not investigated. The geriatric ward substudy [15] showed that all ESBL colonisation and infection detected in the Caulfield Hospital geriatric wards could be traced to nosocomial acquisition in the referring hospital (Alfred Hospital), and identified likely transmission of a *bla*_CTX-M-15_ plasmid between the two most common ESBL strains, ST323 and ST29.

Here we use the full set of KASPAH WGS data to gain a deeper understanding of the burden of 3GCR *K. pneumoniae* infections in the hospital network. We first investigate the ESBL genes and plasmids responsible for the 3GCR phenotype and screen for evidence of plasmid transmission between strains (utilising data on ESBL *E. coli* from the ICU for comparison [16]) using a simple and reproducible method. We explore the contribution of ESBL plasmid transmission to infection burden during the year-long KASPAH study and investigate the long-term persistence of horizontally transferred ESBL plasmids by sequencing 3GCR clinical isolates during 4 years of follow-up (beginning 3 years after the primary study).

## Methods

### Ethical approval

The primary study was approved by the Alfred Health Human Research Ethics Committee (AHHREC), Project numbers #550/12 (19 February 2013) and #526/13 (10 December 2013). Analysis of clinical isolates 2017–2020 was approved by AHHREC, Project number #371/19 (2 July 2019).

### Specimen collection

The primary KpSC collection comprised 440 *Klebsiella pneumoniae*, *Klebsiella variicola* and *Klebsiella quasipneumoniae* isolated at the Alfred Hospital Microbiological Diagnostic Laboratory from patients at hospitals in the Alfred Health network, Melbourne, during a prospective surveillance study (KASPAH) between April 2013 and March 2014 (Additional file 1: Table S1) [13–15]. Gut carriage (colonising) isolates (*n* = 108) were cultured from rectal screening swabs in either the Alfred Hospital ICU (33% of patients screened) [14] or Caulfield Hospital geriatric wards (31% screened) [15]. Eligible ICU patients were adults (aged ≥ 18 years) and expected to spend ≥ 3 days in the ICU. Baseline rectal and throat screening swabs were taken, with follow-up swabs taken every 5–7 days after baseline for the duration of their ICU stay and up to 4 days after transfer from ICU to a different ward. Eligible geriatric patients were adults ≥ 50 years admitted to two geriatric medicine wards at Caulfield Hospital. Rectal and screening swabs were taken at recruitment. Isolates from the carriage cohort came from a total of 102 patients (including 14 with clinical infection). Infection isolates (*n* = 332) represent all KpSC clinical isolates from the Alfred Health network (four hospitals total) that were identified by standard diagnostic protocols across the study period from a cohort of 303 patients [13]. Full KASPAH study protocols are reported elsewhere [13–15]. In January to March 2014, 3GCR Gram-negative carriage and infection isolates were also collected from Alfred Hospital ICU patients as described above and in [16], and those identified as Enterobacteriaceae were included here (*n* = 74)*.* All isolates were subjected to antimicrobial susceptibility testing using Vitek2 GN AST cards (bioMérieux) and interpreted using EUCAST cut-offs (2020).

As some patients were represented by multiple isolates, we de-replicated our isolate collection to retain unique patient/sequence type/body site combinations, to ensure we had only one representative of each patient/sequence type/body site isolate for downstream analysis (see column ‘episode_status’ in Additional file 1: Table S1 for the designation of each isolate). Each of these unique combinations was designated as either a carriage episode (gut and throat swab surveillance isolates) or infection episode (all other specimen types designated as infection by standard diagnostic protocols). The onset of each infection episode was classified as community-acquired, healthcare-associated or nosocomial based on the definitions in [13]. Briefly, community-acquired episodes were isolated either from outpatients or from inpatients on days 0, 1 or 2 of the current hospital admission, in individuals with no recorded contact with the Alfred Health Network in the previous 12 months. Nosocomial episodes were isolated ≥ 3 days after the current inpatient admission or within 1 month of a previous admission. Healthcare-associated episodes were those that did not meet the criteria for nosocomial episodes, but had some recorded contact with the Alfred Health Network in the previous 12 months.

To determine whether KpSC lineages harbouring a plasmid of interest (Plasmid A) during the primary study period persisted 3–6 years later, we interrogated all 3GCR KpSC clinical isolates identified by the same diagnostic laboratory between March 2017 and December 2020 (*n* = 418, identified as *K. pneumoniae* or *K. variicola* by MALDI-TOF (Bruker) and resistant to ceftriaxone (MIC ≥ 2 mg/mL)).

### Whole-genome sequence (WGS) analysis

DNA was extracted and sequenced using the Illumina platforms as previously reported [13–16]. A subset (*n* = 70) of ESBL + isolates from the KASPAH study were selected for additional sequencing via the Oxford Nanopore Technologies MinION platform as described previously [17], to generate completed plasmid sequences. These included at least one representative of each unique combination of multi-locus sequence type (ST) and ESBL gene. Representatives of *bla*_CTX-M-15_-positive KpSC STs identified in the 2017–2020 follow-up period were also subjected to MinION sequencing. Genomes were assembled using Unicycler v0.4.7 [18] and annotated with Prokka v1.14 [19]. Completed genome assemblies were deposited in GenBank and Illumina read sets in the NCBI Sequence Read Archive (accessions in Additional file 1: Table S1). STs were identified for each genome using Kleborate v1.1 [20] for KpSC, and mlst v2.19 [21] for *Escherichia*. Acquired AMR genes were detected using Kleborate v1.1 [20] (note that the chromosomally encoded *oqxAB*, *fosA* and *bla*_SHV_ genes are intrinsic core genes and not counted as acquired AMR genes).

### Identification and comparison of ESBL/carbapenemase plasmids

For each ESBL or carbapenemase gene identified in multiple genomes, the closed, completed plasmid sequences harbouring them were compared pairwise using two similarity metrics: (i) Mash similarity [21] and (ii) gene content Jaccard similarity. Mash similarity was determined by comparing nucleotide sequences using Mash v2.1.1 [22], to calculate mash distance, mash similarity was taken as 1 − mash distance. Gene content similarity was calculated as the Jaccard similarity of gene homologs (i.e. *J* = genes in common/total genes in either plasmid) identified using Roary v3.12 [23]. Pairs of plasmids with Mash similarity ≥ 0.98 and gene content similarity ≥ 0.8 were designated the same plasmid (thresholds determined empirically, see Additional file 2: Fig. S1). We performed visual comparisons of plasmid pairs that were considered the same to ensure the empirically derived thresholds were sensible. Plasmid replicon markers and MOB genes were detected using Mobtyper v1.4.9 [24]. Plasmid copy numbers were estimated by Unicycler, which estimates copy number by dividing read depth across each contig to the median depth across the chromosome [18].

For ST323, ST29 and ST347 genomes with no long-read sequencing data available, and the *n* = 418 genomes in the 2017–2020 follow-up collection, we used read mapping to determine the presence of Plasmid A, represented by pINF329. Illumina reads were mapped to pINF329 (accession LR890241) using RedDog [25] to determine coverage and depth of the plasmid sequence as described previously [26]. Genomes were considered Plasmid A-positive if they had ≥ 80% mapping coverage and < 10 single nucleotide variants (SNVs) compared to the pINF329 reference sequence (*n* = 133 genomes). For incomplete genomes in known Plasmid-A carrying STs which were *bla*_*CTX-M-15*_-positive but Plasmid A-negative, we inferred that *bla*_*CTX-M-15*_ was located on the chromosome.

### Phylogenetic analyses

We generated core-genome phylogenies for Plasmid A, and chromosomes of *K. pneumoniae* ST323 (*n* = 40), ST29 (*n* = 17), ST2856 (*n* = 10) and *K. variicola* ST347 (*n* = 54). Illumina reads were mapped to completed reference sequences generated from study isolates (pINF329 for Plasmid A, accession LR890241; INF018 for ST323, accession LR890493; INF250 for ST29, accession LR890374; KPN342 for ST2856, accession CP089384; INF345 for ST347, accession LR890399) using RedDog to identify SNVs as described above. For additional context, chromosome SNV alignments were supplemented with genomes of the same ST from public isolate collections [20] (ST323, *n* = 6; ST29, *n* = 9; ST347, *n* = 3; see Additional file 3: Table S2 for details of outgroup genomes). The resulting SNV alignments were each filtered to include only SNV sites with allele calls in 100% of genomes. The resulting alignments (ST323 = 7450 SNVs; ST29 = 17,430 SNVs; ST2856 = 13 SNVs; ST347 = 53,093 SNVs) were subjected to maximum likelihood phylogenetic inference using RAxML v8.2.9 [27] with a general time-reversible substitution model. We generated a Plasmid A phylogeny by mapping all genomes belonging to these STs to the pINF329 reference sequence (accession LR890241) using RedDog as described above. Genomes positive for Plasmid A (*n* = 133) were included in the Plasmid A phylogeny, constructed using RAxML v8.2.9 as outlined above (alignment length 63 SNVs).

## Results

Year-long surveillance of all KpSC clinical infection (hospital-wide) and carriage (ICU only) isolates identified infections in 303 patients and colonisation in 102 (including 14 with clinical infection) [14]. WGS identified ESBLs in 47 (15%) infections and 10 (10%) carriage episodes, representing 49 unique patients. Additional screening for ESBL + Enterobacteriaceae in ICU patients during the last 3 months of the study identified 25 non-KpSC isolates for comparison (*n* = 24 *Escherichia coli*, *n* = 1 *Escherichia marmotae*) representing 6 infection and 14 rectal carriage episodes [16].

**Fig. 1:**
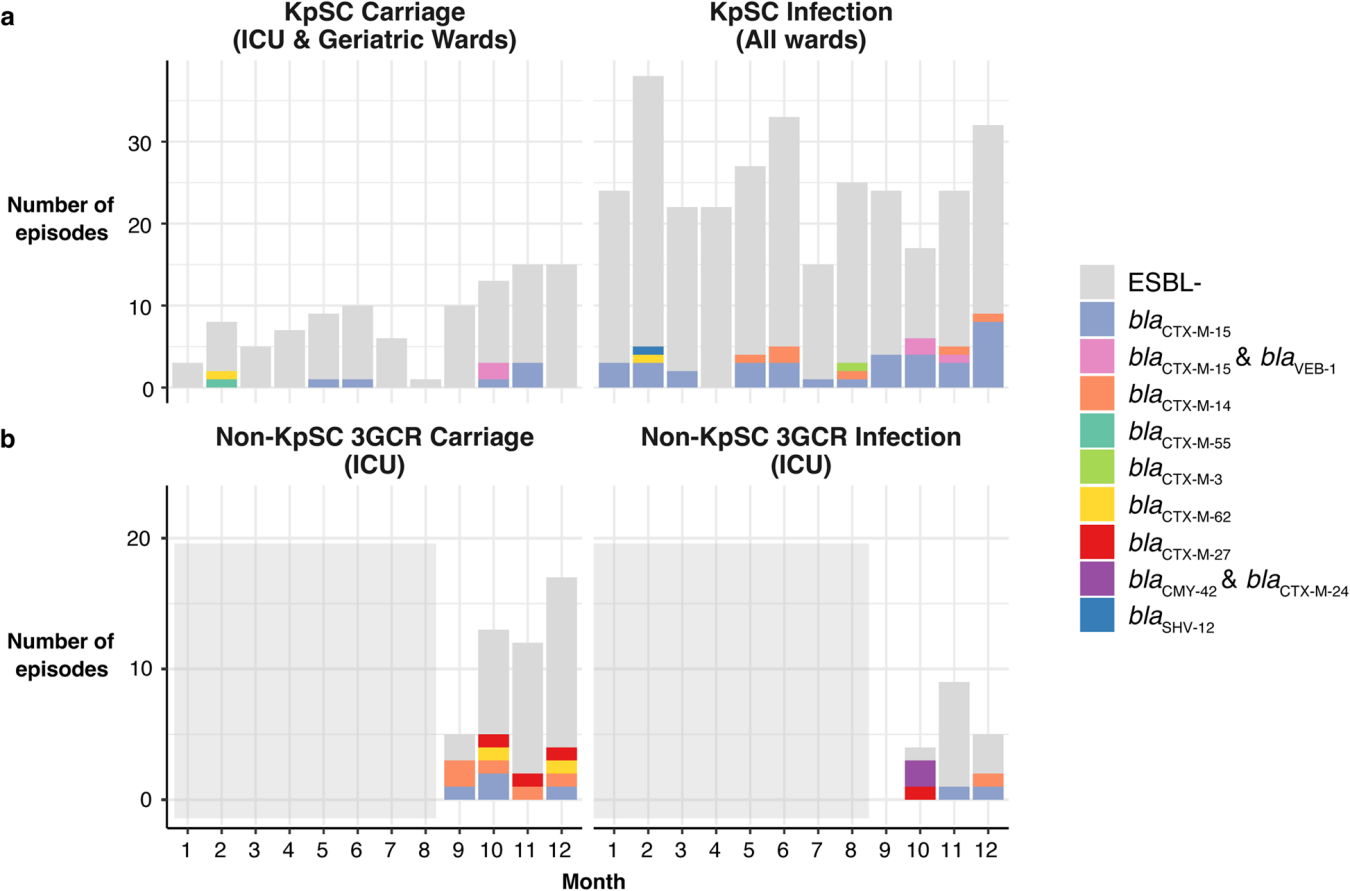
Number of carriage and infection episodes detected per month during the year-long KASPAH surveillance study. **a** KpSC isolates (regardless of 3GCR status); **b** 3GCR non-KpSC Enterobacteriaceae (collected in ICU and during the final 3 months only). Bars are coloured by acquired ESBL gene, as per legend. Light grey boxes in panel **b** indicate no data available for that time period

**Fig. 2:**
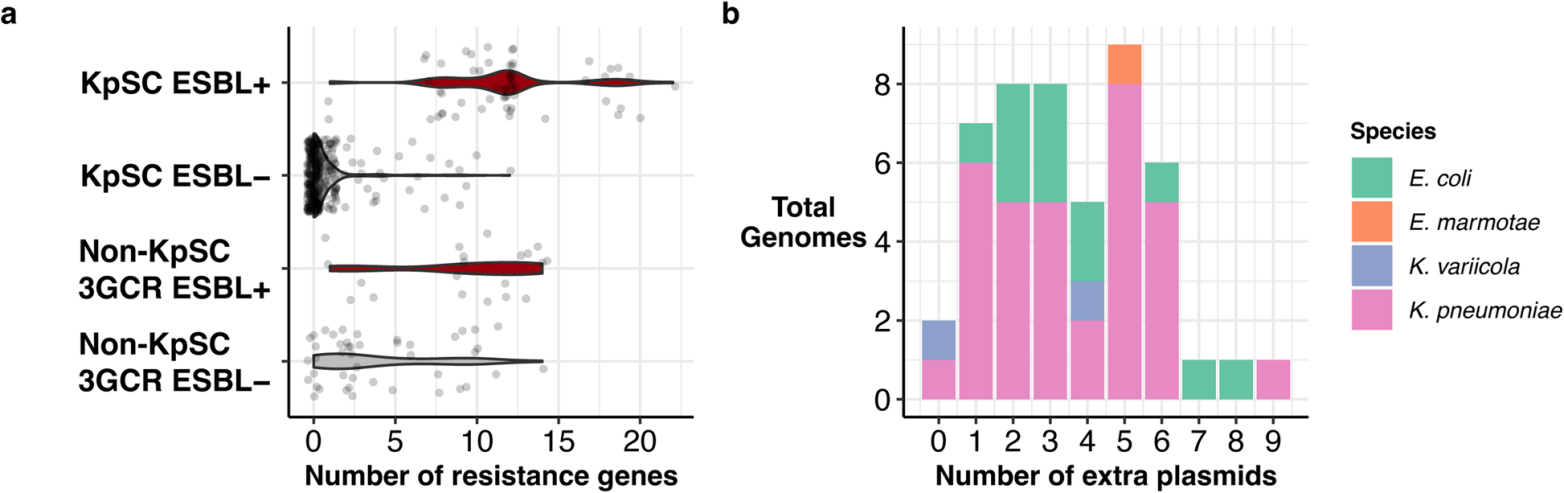
Acquired AMR gene burden and plasmid burden. **a** Distribution of acquired AMR genes across isolates collected in the year-long KASPAH study, stratified by sample group (KpSC, capturing both 3GCR and susceptible isolates; 3GCR, capturing only 3GCR isolates of non-*Klebsiella* Enterobacteriaceae species) and acquired ESBL status. Violin plots are coloured by ESBL status (ESBL + , red; ESBL − , grey). **b** Histogram showing the distribution of non-ESBL plasmid counts for ESBL plasmid-positive genomes. Bars are coloured by species as per legend

All 104 ESBL + isolates were confirmed 3GCR (*n* = 79 KpSC and *n* = 25 *Escherichia*), displaying ceftriaxone MIC > 2 mg/L. Seven different acquired ESBL genes were detected in the KpSC isolates; *bla*_CTX-M-15_ was the most common, present in 85% of all KpSC ESBL + genomes (*n* = 68/79) (Fig. 1a). All KpSC genomes carrying *bla*_VEB-1_ also carried *bla*_CTX-M-15_. Six acquired ESBL genes were detected in the *Escherichia* isolates; again *bla*_CTX-M-15_ was the most common (*n* = 9, 37% of ESBL + *Escherichia*) (Fig. 1b). Twelve KpSC isolates (all ESBL +) carried carbapenemase genes: *bla*_OXA-48_ (n = 8 genomes, five episodes, all *K. pneumoniae* ST231 and also carrying *bla*_CTX-M-15_) and *bla*_IMP-4_ (*n* = 4 genomes, two episodes, all *K. pneumoniae* ST340 and also carrying *bla*_CTX-M-15_). ESBL + isolates harboured a significantly higher burden of acquired AMR genes than ESBL − ones, amongst both KpSC (median 12 vs 0, *p* < 10^−15^ using Wilcoxon rank-sum test, one representative per episode) and *Escherichia* (median 10 vs 2, Wilcoxon test, *p* = 5.4 × 10^−5^) (Fig. 2a).

### ESBL plasmid diversity

To determine the genetic context of ESBL genes, we generated complete closed reference sequences for *n* = 67/104 ESBL + genomes (64%), including at least one for each unique combination of ESBL gene and species/ST, representing *n* = 48/77 of all ESBL episodes (62%). Most of the completed KpSC genomes (*n* = 48/52, 92%) carried a plasmid-borne ESBL gene (including *n* = 14 with an additional chromosomal copy); the rest (*n* = 4/52, 8%) carried a chromosomal ESBL gene only (*bla*_CTX-M-15_). Similarly, amongst *Escherichia* genomes, 87% (*n* = 13/15) carried a plasmid-borne gene (including *n* = 2 with an additional chromosomal copy) and 13% (*n* = 2/15) carried a chromosomal ESBL gene only (*bla*_CTX-M-15_).

Using pairwise similarity scores to compare all 61 completed ESBL plasmid sequences (see the ‘Methods’ section), we identified 25 distinct plasmids. Those found in only a single genome were labelled with their host isolate, and those found in ≥ 2 genomes were labelled Plasmid A, Plasmid B, etc. Each ESBL plasmid was restricted to either KpSC (*n* = 12) or *Escherichia* (*n* = 13) (Table 1). The majority (*n* = 17, 68%) were IncF type plasmids, ranging in size from 45 to 245 kbp with estimated copy number 1.3–3 per cell, and accounting for 70% of the *bla*_CTX-M_ genes that were identified (Table 1, Additional file 4: Table S3). Other notable ESBL plasmids included two IncN plasmids carrying *bla*_CTX-M-62_ (both copy number ~ 5x; one 59 kbp, the other 102 kbp and also harbouring an IncR replicon marker), and a small (10 kbp) high copy number (16x) plasmid carrying *bla*_CTX-M-14_ in *E. coli* ST48 (Table 1, Additional file 4: Table S3).

**Table 1:** Unique ESBL plasmids found in this study

Plasmid	ESBL	#Pt	#G	Organisms	Median size (bp)	Inc type(s)	MOB	Median copy number	# AMR classes (genes)
***KpSC ESBL Plasmids***									
Plasmid A	*bla*_CTX-M-15_	18	39	Kp (4 STs) Kv ST347	243,577	FIB, FII	F	1.30	3–5 (5–8)
Plasmid B	*bla*_CTX-M-15_ *bla*_VEB-1_	4	6	Kp ST231	128,238	C (ST3)	H	1.49	6 (12)
Plasmid C	*bla*_CTX-M-15_	4	4	Kp ST491	190,324	FIA, FII	F	1.49	6 (6)
Plasmid D	*bla*_CTX-M-15_	3	8	Kp ST340	89,345	FIA, R	-	2.20	5 (9)
pINF044	*bla*_CTX-M-15_	1	1	Kp ST437	227,214	FIB, FII	F	1.07	6 (9)
pINF161	*bla*_CTX-M-14_	1	1	Kp ST661	89,062	FIIA	P	1.15	0 (0)
Plasmid E	*bla*_CTX-M-14_	3	3	Kp ST17	214,317	FIB, FII	F	1.24	7 (10–12)
Plasmid F	*bla*_CTX-M-14_	2	2	Kp ST3062	159,592	FIB, FIIA, FII, FIA	F	2.58	5 (8)
Plasmid G	*bla*_CTX-M-62_	2	2	Kp ST15	102,016	N, R	P, F	5.97	8 (11)
pINF223	*bla*_CTX-M-3_	1	1	Kp ST3072	60,529	FII	P	2.98	2 (2)
pKSB1_1B	*bla*_CTX-M-55_	1	1	Kp ST1213	113,743	I2	P	1.30	0 (0)
pINF058	*bla*_SHV-12_	1	1	Kv ST3076	279,735	H	H	1.55	8 (8)
***Escherichia ESBL plasmids***									
pMSB1_1A	*bla*_CTX-M-15_	1	1	Ec ST617	173,787	FIB, FIIA, FII, FIA	F	1.53	7 (10)
pMSB1_9I	*bla*_CTX-M-15_	1	1	Ec ST10	44,618	FIIA, FII	F	1.30	0 (0)
pMINF_8D	*bla*_CTX-M-15_	1	1	Ec ST10	115,278	unknown	none	1.72	0 (0)
pMINF_1D	*bla*_CTX-M-15_	1	1	Ec ST141	125,802	FIB, FIIA, FII, FIA	F	1.55	1 (1)
Plasmid H	*bla*_CTX-M-14_	2	2	Ec ST48	10,057	none	none	15.81	2 (2)
pMINF_2E	*bla*_CTX-M-14_	1	1	Ec ST602	207,369	FIB, FIIA, FII	P, F	1.58	0 (0)
Plasmid I	*bla*_CTX-M-14_	2	2	Ec ST38	70,350	FIIA, FII	F	2.71	0 (0)
pMSB1_5C	*bla*_CTX-M-14_	1	1	Em ST3727	70,361	FIIA, FII	F	2.63	0 (0)
Plasmid J	*bla*_CTX-M-27_	2	3	Ec ST131	112,822	FIB, FIIA, FII, FIA	F	1.95	0 (0)
Plasmid K	*bla*_CTX-M-27_	1	2	Ec ST38	160,498	FIB, FIIA, FII, FIA	F	1.91	6 (9)
pMINF_9A	*bla*_CTX-M-27_	1	1	Ec ST648	139,312	FIB, FIIA, FII, FIA	F	1.79	5 (7)
Plasmid L	*bla*_CTX-M-62_	2	2	Ec ST176	59,604	N	F	4.83	5 (5)
Plasmid O	*bla*_CMY-42_	2	2	Ec ST354	47,582	I1	P	2.05	0 (0)

*#Pt* Number of patients, *#G* Number of genomes (completed and draft), *Kp K. pneumoniae*, *Kv K. variicola*, *Ec E. coli*, *Em E. marmotae*

Ten of 12 KpSC ESBL plasmids carried additional AMR genes (median 8.5 AMR genes per plasmid); however, only half (*n* = 6/13) of the *Escherichia* ESBL + plasmids carried additional AMR genes (median 6 per plasmid) (Table 1, Additional file 4: Table S3). Nearly all (*n* = 45/48, 93%) of the completed ESBL plasmid-carrying KpSC genomes carried at least one additional plasmid (range 0–9 plasmids), and 70% carried at least three (Fig. 2b). The majority of additional plasmids did not carry AMR genes, except for two STs carrying carbapenemase plasmids: *K. pneumoniae* ST340 with *bla*_IMP-4_ (*n* = 4 isolates) and *K. pneumoniae* ST231 with *bla*_OXA-48_ (*n* = 8 isolates) (Additional file 4: Table S3). All of the completed ESBL plasmid-carrying *E. coli* genomes carried at least one additional plasmid (range 1–8) (Fig. 2b), the majority of which lacked AMR genes (*n* = 8/13, 62%).

### Plasmid transmission network

**Fig. 3:**
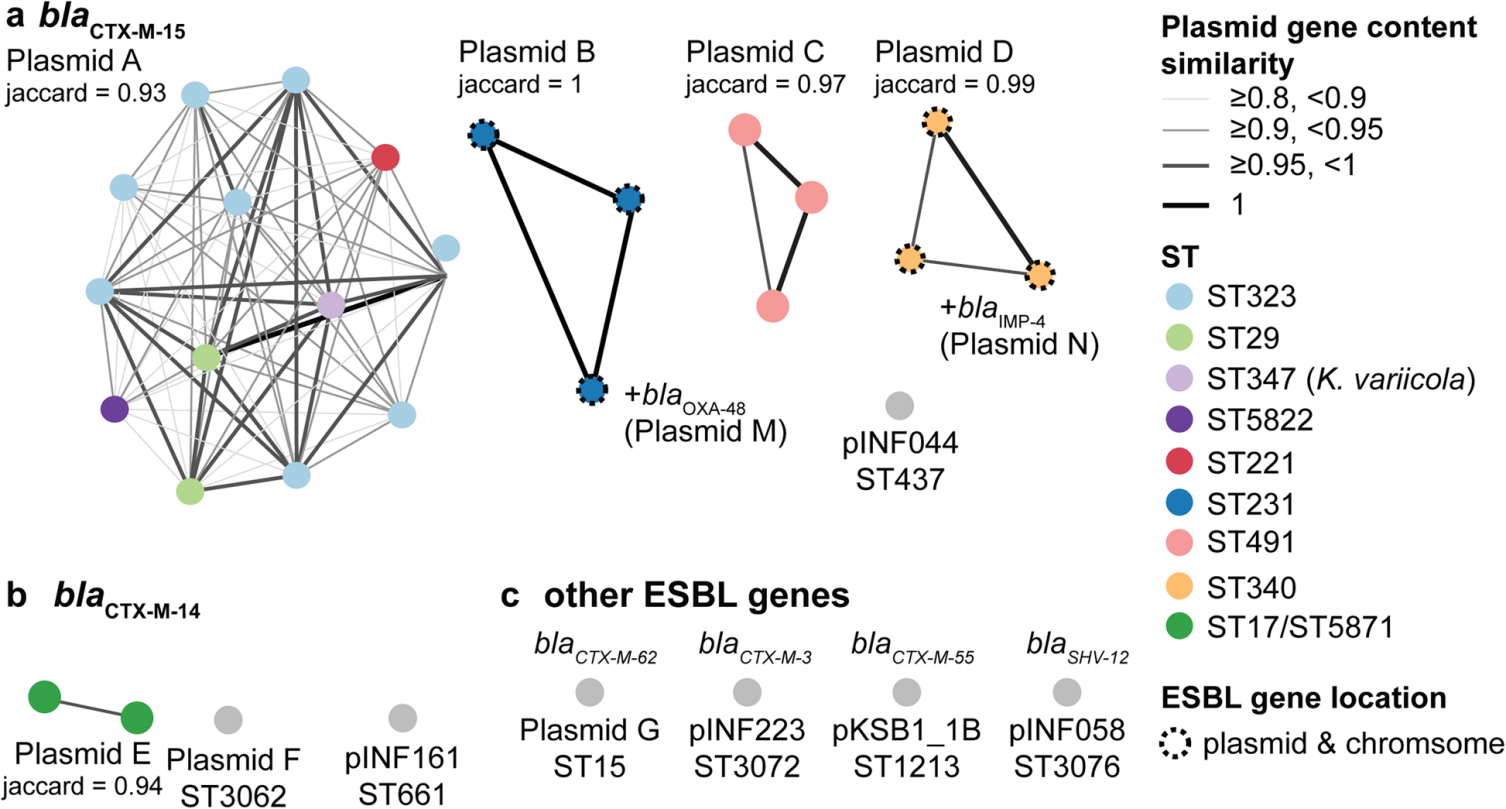
ESBL plasmid cluster network for all completed KpSC ESBL + genomes. **a** *bla*_CTX-M-15_ plasmids; **b** *bla*_CTX-M-14_ plasmids; **c** other detected ESBLs. Each node represents a completed genome (from one representative isolate per patient), coloured by the bacterial host cell ST as per legend. Dashed outline surrounding dot indicates if the ESBL is carried on both a plasmid and the chromosome. Nodes are connected if they share a similar plasmid sequence, defined as mash similarity ≥ 0.98 and gene content similarity ≥ 0.8 (see Additional file 2: Fig. S1); line colour and width indicate plasmid gene content similarity (as per legend). Clusters comprising ≥ 2 genomes are labelled with the median jaccard gene similarity score. ‘ + *bla*_OXA-48_ (Plasmid M)’ and ‘ + *bla*_IMP-4_ (Plasmid N)’ indicate STs that carry an additional carbapenemase plasmid (details in Additional file 4: Table S3)

**Fig. 4:**
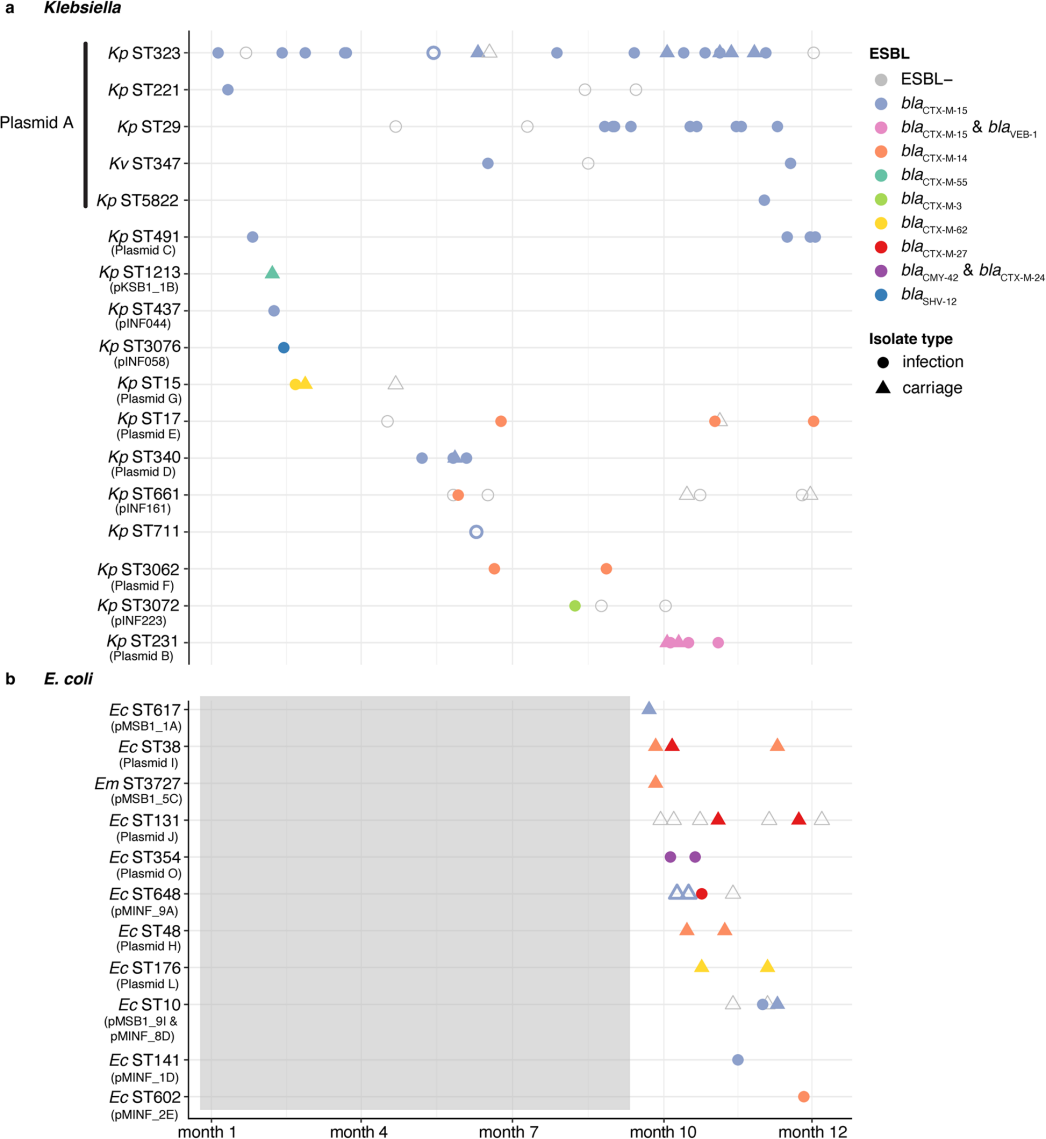
Timeline of ESBL + infection and carriage episodes. **a** ESBL + *Klebsiella* infection episodes (all wards) and carriage episodes from the ICU. Each episode is represented by a single isolate, grouped in rows by the isolate ST. Colours indicate the ESBL enzyme encoded in the genome (as per legend), and shapes indicate whether the episode was carriage (circle) or infection (triangle). Presence of the ESBL plasmid most commonly associated with the ST is indicated by filled shapes (with plasmid name listed under ST on the y-axis). ESBL genes on the chromosome rather than plasmid are indicated by shapes with no fill and a coloured outline. Kp, *K. pneumoniae*; Kv, *K. variicola.* **b** ESBL + *Escherichia* infection (ICU-only) and carriage episodes from the ICU. Each episode is represented by a single isolate, shapes and colours as per panel **a**. Grey box indicates that no data was available for this period for the *Escherichia* STs. Ec, *E. coli*; Em, *E. marmotae*

We used the completed genomes to investigate the evidence for nosocomial transmission of ESBL plasmids between distinct KpSC strain backgrounds (defined by ST), by constructing a network in which nodes represent ESBL + plasmids identified in KpSC (Fig. 3). The vast majority of unique ESBL plasmids were detected in either a single genome (*n* = 13/25) or a small number of genomes of a single ST (*n* = 11/25, 2–8 genomes each) (Table 1, Figs. 3 and 4a). However, Plasmid A (which carried *bla*_CTX-M-15_) was identified in 41 isolates belonging to four *K. pneumoniae* STs (ST323, ST29, ST5822, ST221) and *K. variicola* (ST347, see Fig. 3). Plasmid A was highly conserved between isolates, with median pairwise gene content similarity 0.93 (range 0.84–1) and median Mash similarity 0.998 (range 0.996–1; see Fig. 3, Additional file 4: Table S3). Median 0 SNVs were detected amongst Plasmid A sequences (19 identical, range 0–54 SNVs), regardless of host chromosomal ST, with only occasional differences in plasmid gene content (Additional file 2: Fig. S2). Taken together, these data support transmission of Plasmid A between multiple *K. pneumoniae* lineages and one *K. variicola* lineage.

We found no evidence of ESBL plasmid transmission amongst *Escherichia* STs from the final 3 months of the study, with each *Escherichia* ST harbouring its own ESBL plasmid (Fig. 4b).

### Dissemination of Plasmid A and contribution to ESBL burden

**Fig. 5:**
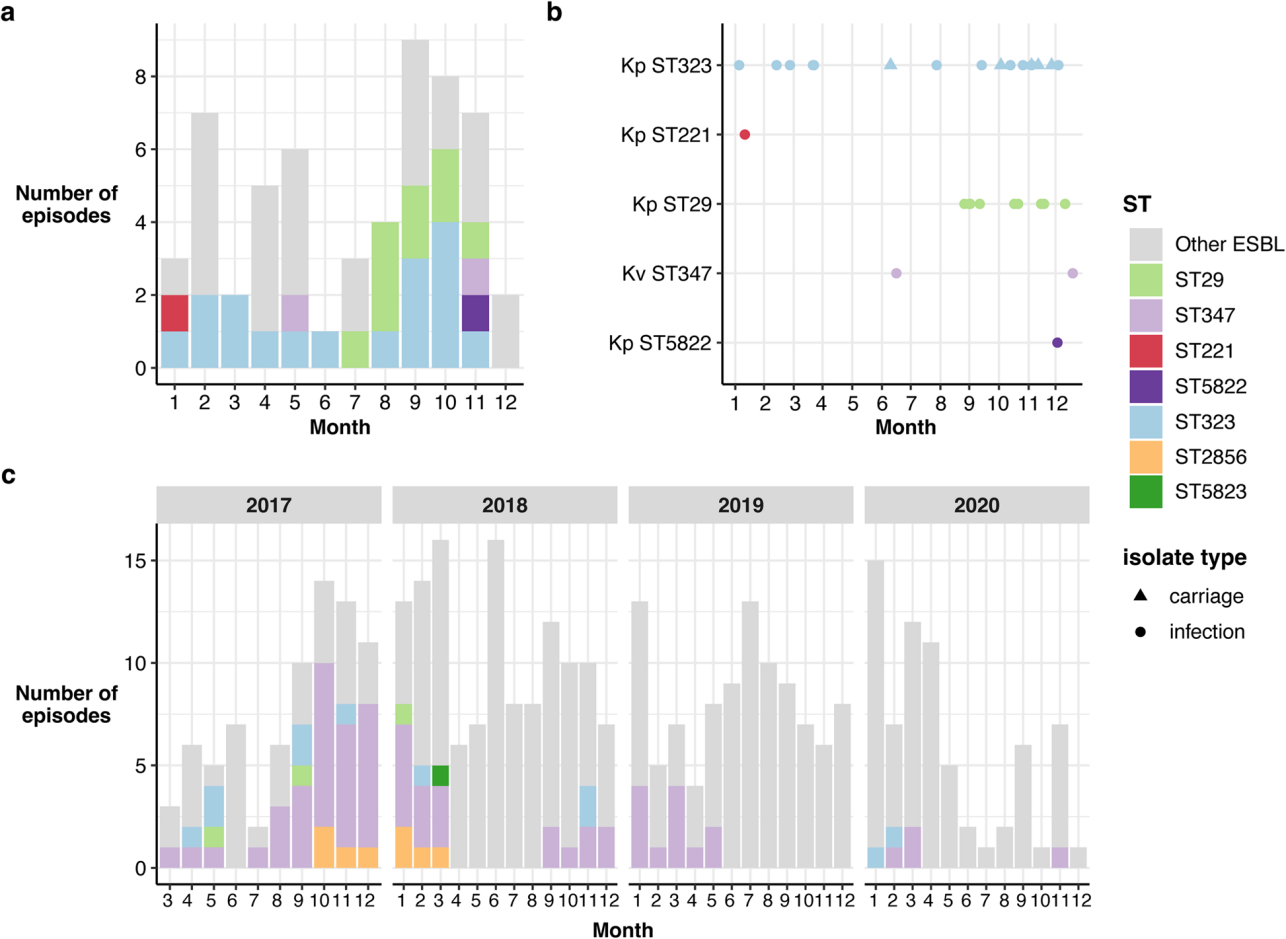
Presence of Plasmid A over time. **a** Bar height indicates total number of ESBL episodes across the initial year-long study period, by month. Bars are coloured by Plasmid A positive ST episodes as per legend, and grey for non-Plasmid A ESBL episodes. **b** Timeline of Plasmid A positive episodes during the initial year-long study period. Each point represents an episode, with colour indicating ST (as per legend) and shape indicating whether the episode was infection (circle) or carriage (triangle). **c** Bar height indicates the total number of 3GCR KpSC episodes per month from March 2017 to December 2020. Bars are coloured by ST as per legend if the episode was positive for Plasmid A

Overall Plasmid A was present in 51% (*n* = 29/57) of KpSC ESBL + episodes (53% of ESBL + infections, *n* = 26/49 unique patients) during the study period. The remaining 49% (*n* = 28/57) of KpSC ESBL + episodes were attributed to plasmids/strains isolated from one patient only (Fig. 5a). *K. pneumoniae* ST323 was the most common host strain for Plasmid A (*n* = 22). Plasmid A-positive ST323 were detected throughout the entire study period and accounted for 29% of ESBL + KpSC episodes (25.5% of infections, 50% of carriage) (Fig. 4a). Of the 16 Plasmid A-positive ST323 episodes, five were health-care associated and nine were nosocomial, whilst only two episodes were considered community-acquired (Additional file 1: Table S1). Plasmid A-positive ST323 isolates from distinct patients (*n* = 17) differed from each other by 0–55 chromosomal SNVs (median 22, see Additional file 2: Fig. S3), consistent with clonal transmission of this strain within the hospital network as previously reported [15].

We hypothesise that Plasmid A was originally hosted in ST323 and subsequently transmitted into four other KpSC STs. ST323 carrying Plasmid A was present throughput the study period, with detection of ST29 and ST347 Plasmid A-positive strains later in the sampling timeframe. Two ST29 isolates collected in months 4 and 7 did not carry Plasmid A (INF206 and INF122, Fig. 4) and were evolutionarily distant from the 11 Plasmid A-positive ST29 genomes recovered in months 8–12 (INF206 = 10,341 core genome SNVs, INF122 = 106 SNVs; Additional file 2: Fig. S3, all of which were either healthcare-associated or nosocomial) [15]. A Plasmid A-negative *K. variicola* ST347 isolate collected in month 8 (INF238, Fig. 4) was distant to subsequent ST347 isolates (35,020 core genome SNVs; Additional file 2: Fig. S3). As we sequenced all clinical isolates during the study period, we hypothesise that it was unlikely that Plasmid A-positive ST29 or ST347 were circulating in our hospital prior to their detection from month 8 onwards, and thus the timing of their emergence suggests transfer of Plasmid A from ST323. Notably, all Plasmid A-positive ST29, ST347, ST221 and ST5822 were either healthcare-associated or nosocomial episodes (Additional file 1: Table S1), consistent with acquisition of either the strain or the plasmid within the hospital setting. Analysis of the Plasmid A backbone sequence showed that there were few variants present across the Plasmid A phylogeny (Additional file 2: Fig. S2). Hence, we hypothesise that the plasmid was acquired in the hospital, and the resulting plasmid-positive strains subsequently transmitted in the hospital.

We detected only a single episode each of ST221 and ST5822 with Plasmid A, i.e. there was no evidence of these Plasmid A-positive strains being transmitted in the hospital. However the data supported nosocomial transmission of Plasmid A-positive ST29 and ST347 strains (median chromosomal SNVs between isolates from different patients = 1 for both STs, range 0–5, *n* = 9 patients for ST29 and *n* = 2 patients for ST347). Assuming all four STs acquired Plasmid A from ST323, these plasmid transmission events and subsequent onward transmission of the resulting ESBL + strains would account for 23% (*n* = 13/57) of KpSC all ESBL + episodes (28% of ESBL + infections) during the 1-year study period, including 54% of those in the last 3 months (Fig. 5a, b).

### Long-term persistence of Plasmid A in diverse strain backgrounds

We screened for Plasmid A in additional WGS data from 3GCR KpSC clinical isolates collected 3–6 years later from the same hospital laboratory (*n* = 418, representing 92% of all 3GCR KpSC clinical isolates during March 2017–Dec 2020). These 418 isolates represented 365 infection episodes (57% urinary tract, 13% bacteraemia, 12% respiratory, 5.8% wound/soft tissue). Plasmid A was identified in 35% of all sequenced 3GCR isolates and 21.2% of all 3GCR infection episodes (24%, 21%, 42% and 8.5% of 3GCR urinary, bacteraemia, wound/soft tissue and respiratory episodes, respectively), representing 81 patients. We observed the same three expanded STs seen in the earlier time period (*n* = 11 ST323, *n* = 3 ST29, *n* = 69 ST347), as well as two additional STs (*n* = 11 ST2856, *n* = 1 ST5823) (Fig. 5c). Phylogenetic analysis of the host chromosomes was consistent with persistence of the plasmid during long-term local clonal expansion of the ST323, ST29 and ST347 host strains (Additional file 2: Fig. S3, median pairwise chromosomal SNVs = 31, 10 and 5 for ST323, ST29 and ST347 respectively). Inspection of completed genome sequences for one genome per ST (KPN029, KPN110, KPN392, KPN342, KPN692) confirmed that the more recent isolates carried Plasmid A, with few structural changes (Additional file 2: Fig. S4a). Plasmid A was most prevalent from March 2017 to March 2018, accounting for 52% of 3GCR episodes (*n* = 62/120, Fig. 5c). From April 2018 onwards, Plasmid A episodes were rare (< 10%), except for a cluster of infections between September 2018 and May 2019 during which time Plasmid A-positive ST347 accounted for 27.5% of 3GCR infections (Fig. 5c).

### Carbapenemase plasmid transmission

Whilst the majority of MDR burden in our hospital was due to ESBLs, we also detected three STs carrying a carbapenemase plasmid in addition to their ESBL plasmid: (i) *K. pneumoniae* ST340 (n = 4 isolates, Additional file 2: Fig. S5a), which carried both an IncFIA/IncR ESBL plasmid harbouring *bla*_CTX-M-15_ (Plasmid D) plus nine other AMR genes and an IncC carbapenemase plasmid harbouring *bla*_IMP-4_ plus five other AMR genes (Plasmid N, Additional file 4: Table S3); (ii) *K. pneumoniae* ST231 (*n* = 8 isolates, Additional file 2: Fig. S5a), which carried an IncC ESBL plasmid harbouring *bla*_CTX-M-15_, *bla*_VEB-1_ and 12 other AMR genes (Plasmid B), plus an IncL/M carbapenemase plasmid harbouring *bla*_OXA-48_ without other AMR genes (Plasmid M, Additional file 4: Table S3); and (iii) *E. coli* ST38 (*n* = 1 isolate, Additional file 2: Fig. S5a) which carried an IncF ESBL plasmid harbouring *bla*_CTX-M-27_ and 9 other AMR genes (Plasmid K), plus the *bla*_OXA-48_ Plasmid M.

These 13 carbapenemase isolates were spread across six individual patients, five of which had multiple isolates taken during their hospital stay (Additional file 2: Fig. S5b). The majority of patients with multiple isolates (*n* = 4/5) were infected or colonised with the same strain (Additional file 2: Fig. S5b). However, patient AH0269 was colonised with two carbapenemase-producing strains, *K. pneumoniae* ST231 and *E. coli* ST38, both harbouring the Inc L/M Plasmid M with *bla*_OXA-48_ (Additional file 2: Fig. S5a,b).

Patient AH0269 was a burns patient who had four *Enterobacteriaceae* carriage isolates plus four infection isolates (Additional file 2: Fig. S5c). This patient was colonised by three unique strains (*K. pneumoniae* ST231 and *E. coli* ST38 detected at 2 and 6 days after ICU admission, and *Pseudomonas aeruginosa* ST357 detected at 2 days after admission), plus one vancomycin-resistant *Enterococcus* (ST17, detected 6 days after admission) (Additional file 2: Fig. S5c). Additionally, *K. pneumoniae* ST231 and *E. coli* ST354 were isolated from wound infections (cultured 1 day after ICU admission) whilst *P. aeruginosa* ST357 and *Burkholderia cenocepacia* were isolated from central venous catheter-associated infections (7 days after admission) (Additional file 2: Fig. S5c). All of the *K. pneumoniae* isolated from patient AH0269 were carbapenem-resistant, harbouring Plasmid M.

The first *E. coli* ST38 isolate (MSB1_7A) was cultured from the day two rectal swab and was lacking Plasmid M (Additional file 2: Fig. S5c). Subsequently, an *E. coli* ST38 isolate that carried Plasmid M (MSB1_3B) was cultured from the day six rectal swab (Additional file 2: Fig. S5c). High-resolution comparison of the two *E. coli* isolates indicated no single nucleotide variants (SNVs) differentiating their chromosomes. Hence, we concluded that Plasmid M was likely transferred from a *K. pneumoniae* ST231 donor to an *E. coli* ST38 recipient within the patient’s gut microbiota. The genome data also suggests that the Plasmid M-positive *K. pneumoniae* ST231 strain was transmitted to four other patients in the ICU (≤ 8 core genome SNVs, 0 plasmid SNVs, Additional file 2: Fig. S5b, also discussed previously [14]). Plasmid M is highly similar to the *bla*_OXA-48_ IncL plasmid previously described in *K. pneumoniae* [28] (NC_019154 is 99% identical with 95% coverage to Plasmid M), which has previously been shown to transmit between multiple species within the *Enterobacteriaceae*, including *K. pneumoniae*, *Enterobacter cloacae*, *Salmonella enterica* and *E. coli*, most frequently within the gastrointestinal tracts of hospital patients [29].

## Discussion

Here, we present a systematic comparison of ESBL strain and plasmid transmission over a year in a single hospital network. Whilst there was no evidence of plasmid transmission for most ESBL + plasmids, Plasmid A was the exception and we speculate that ST323 was the original donor strain. As we have only sampled KpSC from a single institution, we cannot be certain that Plasmid A transmission events occurred within our specific hospital and note that ESBL + ST323 has been reported in other Melbourne hospitals [8]. However, it is quite likely that the plasmid transmission events occurred in a hospital setting under selective pressure from antibiotics, since ESBL KpSC is known to be relatively rare outside of the hospital environment in Australia and other high-income countries [30, 31]. Additionally, whilst our sample includes all clinical infection isolates, surveillance screening for carriage was only performed in the ICU and geriatric wards, and we did not perform any environmental sampling. This means that we are unable to specifically determine if or where Plasmid A transmission may have occurred in our hospital (e.g. within the guts of patients outside of the geriatric ward/ICU, or within other environments around the hospital, or outside the hospital). We also did not extensively sample other species within the Enterobacteriaceae (aside from the final 3 months of the initial study period which included all multi-drug-resistant Enterobacteriaceae from the ICU) that may have been harbouring Plasmid A.

Our results indicate that Plasmid A is stable in at least some strain backgrounds, with three out of six putative recipient strains going on to spread in the hospital in parallel with the dissemination of Plasmid A-positive ST323. Compared with the other ESBL + plasmids identified in this study, which showed no evidence of transfer between strains, Plasmid A likely has properties that (i) make it efficient at horizontal transfer and/or (ii) reduce the fitness cost and thus facilitate its stable maintenance. Plasmid A is very similar to the IncFIB/IncFII *bla*_CTX-M-15_ plasmid found in the globally disseminated *K. pneumoniae* clone ST307 (pKPN-307, median Mash distance 0.99, 70% gene similarity to Plasmid A, with some differences in gene order; Additional file 2: Fig. S4b), which has been stably maintained for decades [32]. pKPN-307 carries five distinct virulence clusters that are hypothesised to aid long-term survival of ST307 outside of the human host [33]; all five of these clusters are conserved in Plasmid A, in addition to heavy metal resistance operons (Additional file 2: Fig. S4c). Other IncFIB/IncFII plasmids detected in the globally distributed *K. pneumoniae* ST258 also carry many of these genes [34], suggesting that they may be a feature of this plasmid background. Understanding what plasmid and/or host strain properties enable stable maintenance of large AMR plasmids within a particular genetic background is vital to improving our understanding of how AMR strains emerge and persist [35].

CTX-M-15 was the most frequent ESBL in our dataset and was always found downstream of the transposase ISEcp1, regardless of plasmid backbone. ISEcp1 with *bla*_CTX-M-15_ is the most common configuration for this ESBL gene, where ISEcp1 provides the promotor required for expression of *bla*_CTX-M-15_, and has been found in multiple plasmid backbones, including IncF plasmids such as Plasmid A [36, 37]. This transposon is clearly active, as we detected integration of ISEcp1 + *bla*_CTX-M-15_ into the chromosomes of 14 isolates that carried *bla*_CTX-M-15_ plasmids (*n* = 1 ST323 with Plasmid A, *n* = 5 ST231 with Plasmid B, *n* = 7 ST340 with Plasmid D and *n* = 1 ST15 with Plasmid G). In ST323, we detected an additional four isolates that no longer carried Plasmid A, but had *bla*_CTX-M-15_ in their chromosome, and we hypothesise that the donor was Plasmid A. However, as we have only completed plasmids from isolates with ESBL enzymes, we were unable to discern the impact that mobilisation of ISEcp1 + *bla*_CTX-M-15_ into ESBL − plasmids might have had in our hospital.

## Conclusions

ESBL infections contribute to increased length of hospital stays, resulting in higher healthcare costs [38]. Models examining genomic surveillance have been shown to reduce overall healthcare costs [39], but have not considered the impact of plasmid transmission on AMR burden. Given that half of all ESBL episodes in the primary 1-year study period, and one-fifth of all 3GCR episodes in the follow-up period 3–6 years later were due to only a few Plasmid A-positive strains, prevention of onward transmission of these strains could have reduced ESBL burden in our hospital. This could also reduce the number of opportunities for Plasmid A to transmit and create novel ESBL + strains. Whilst our study focused on ESBL plasmids, those carrying carbapenemases transfer via the same mechanisms and show similar transmission dynamics. Like ESBL plasmids, some carbapenemase plasmids have been found to transmit amongst the Enterobacteriaceae in hospital environments [29, 40], and in particular, *Klebsiella* has been known to harbour multiple ESBL and carbapenemase plasmids in a single cell [41, 42]. This study highlights the need for improved approaches to detect plasmid transmission using genomic data within an infection control framework.

## Supplementary Information

**Additional file 1: Table S1.** Details of all isolates used in this study.

**Additional file 2: Supplementary Figs. S1, S2, S3, S4, S5**.

**Additional file 3: Table S2.** Details of outgroup genomes used for chromosomal ST323, ST29 and ST347 trees.

**Additional file 4: Table S3.** Details of unique ESBL and carbapenemase plasmids found in this study. Kp – *K.pneumoniae*; Kv – *K. variicola*; Ec– *E. coli*; Em – *E. marmotae*.

## Data Availability

The data generated in this study are available on NCBI; please see Additional file 1: Table S1 for individual read and assembly accessions.
