# Supplementary material for: ESBL plasmids in Klebsiella pneumoniae: diversity, transmission and contribution to infection burden in the hospital setting
Source: Genome Med. 2022 Aug 23;14:97. doi: 10.1186/s13073-022-01103-0 (PMC9396894; doi:10.1186/s13073-022-01103-0)
Supplement: Supplementary file 2 — Additional file 2: Supplementary Figs. S1, S2, S3, S4, S5. [file 13073_2022_1103_MOESM2_ESM.pdf]

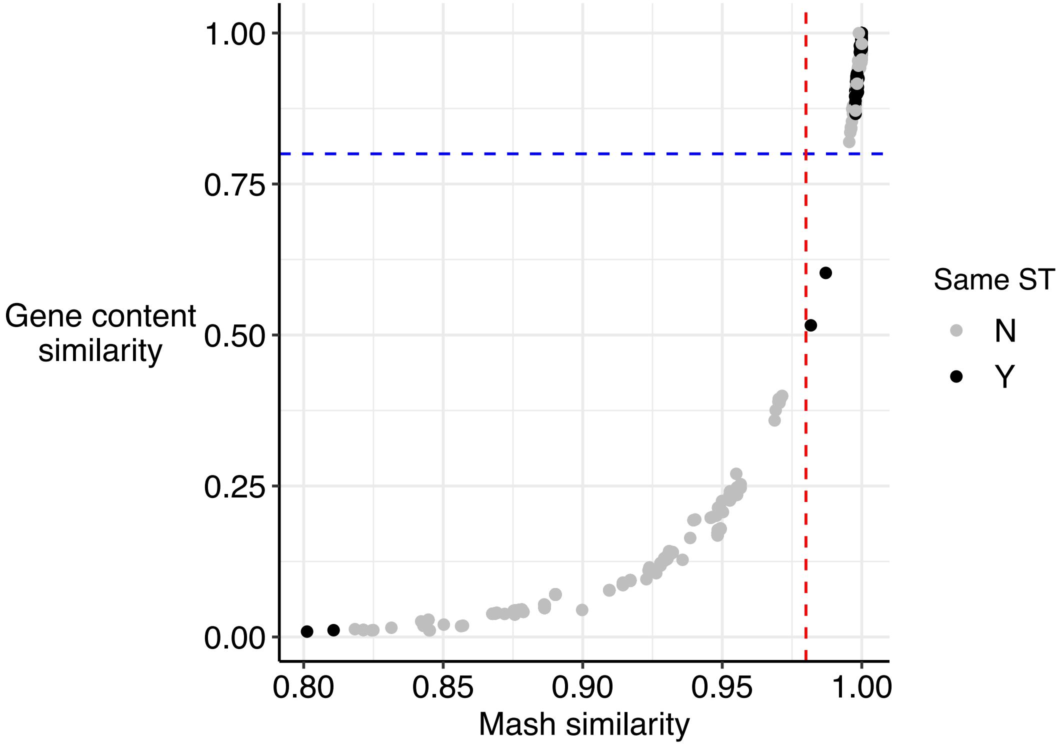

Supplementary Figure 1: Pairwise comparison of *bla*$_{\text{CTX-M-15}}$ plasmid sequences by pairwise mash similarity and jaccard gene content similarity. Each dot represents a pairwise comparison, dots are coloured black if the pair come from the same ST, and grey if they do not. The red dotted line indicates the mash similarity cutoff (0.98), and the blue dotted line the gene content similarity cutoff (0.80). Plasmid sequence pairs whose values were above both cutoffs (upper right corner of the graph) were considered the same plasmid.

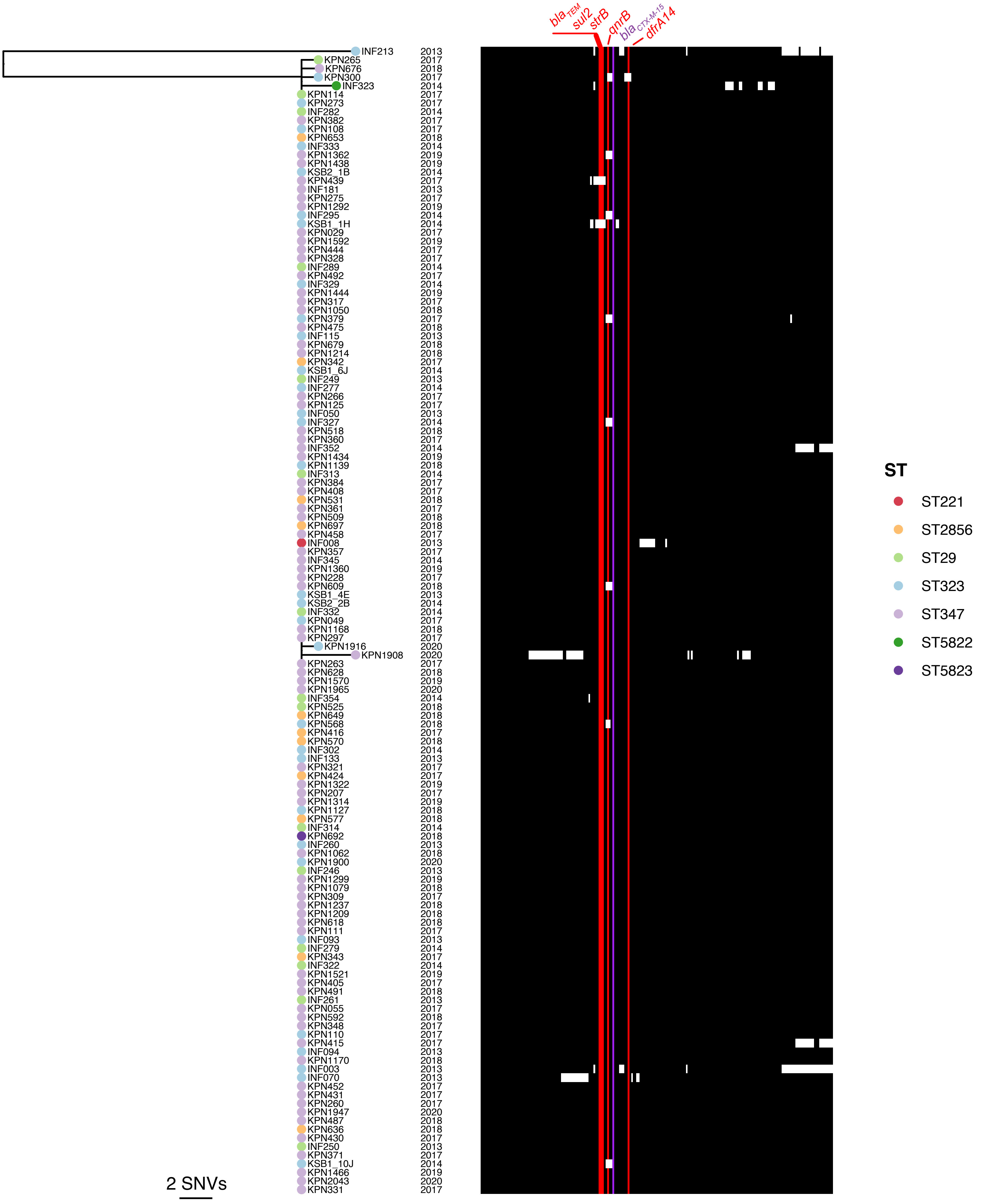

Supplementary Figure 2: Maximum likelihood phylogeny of Plasmid A. Phylogeny is midpoint rooted. Tips are coloured by ST as per legend. Year of isolation is indicated next to each tip. The heatmap shows the presence of each gene on Plasmid A, with a coloured square indicating presence, and white indicating absence. AMR genes found on Plasmid A are indicated by red boxes, with *bla*$_{CTX-M-15}$shown in purple.

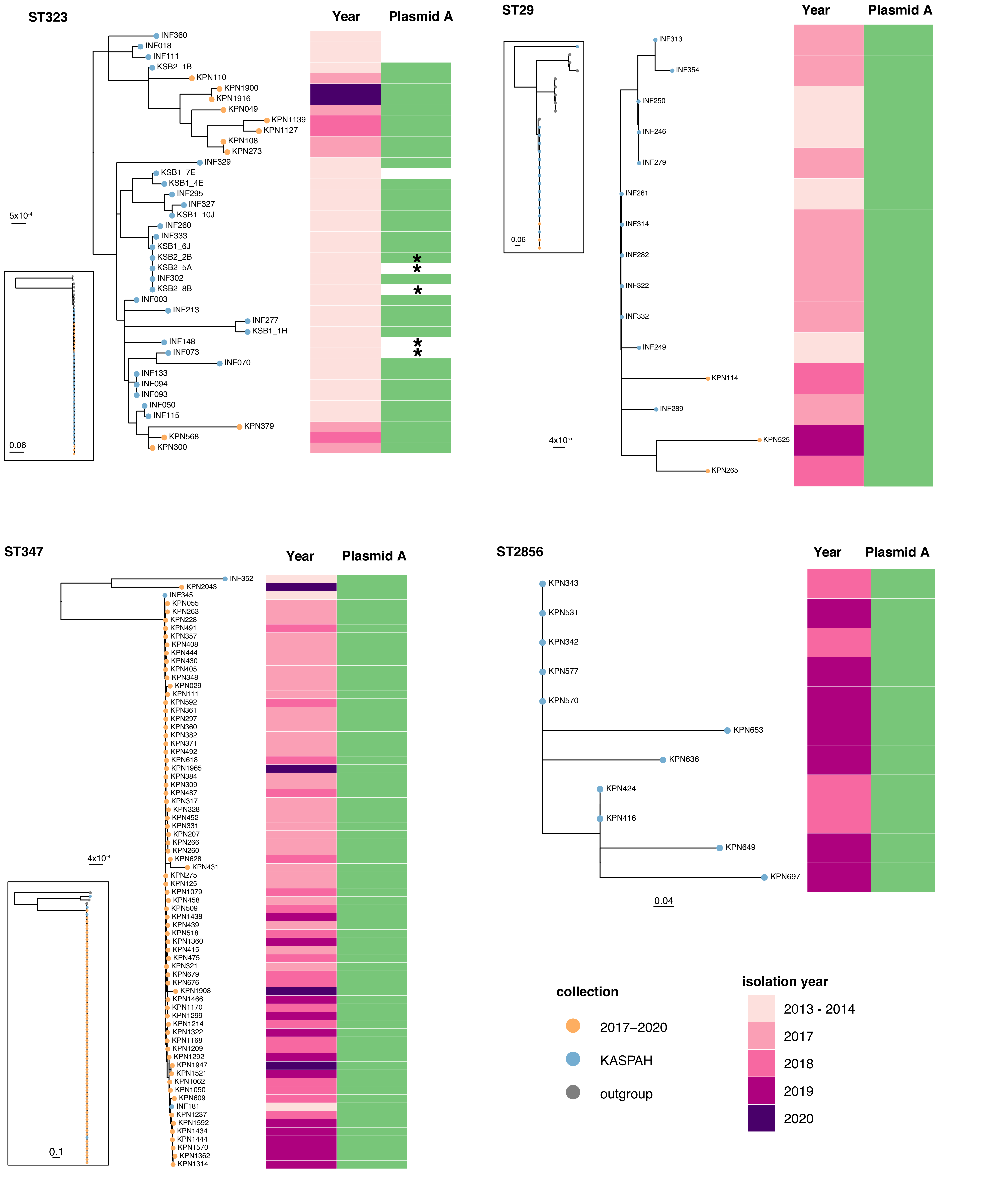

Supplementary Figure 3: Maximum-likelihood phylogenies of genomes from the four major STs that carried Plasmid A in KpSC.
ST323, ST29 and ST347 phylogenies are outgroup rooted using publicly available genomes; ST2856 phylogeny is midpoint rooted as no outgroup genomes were found. Inset trees show full tree, main trees show only the outbreak genomes from this study. Tips of the trees are coloured by collection. Column 1 of the heatmap indicates year of isolation and presence (green) or absence (white) of Plasmid A in column 2. Black stars next to isolates indicate the presence of a chromosomal copy of *bla*$_{CTX-M-15}$.

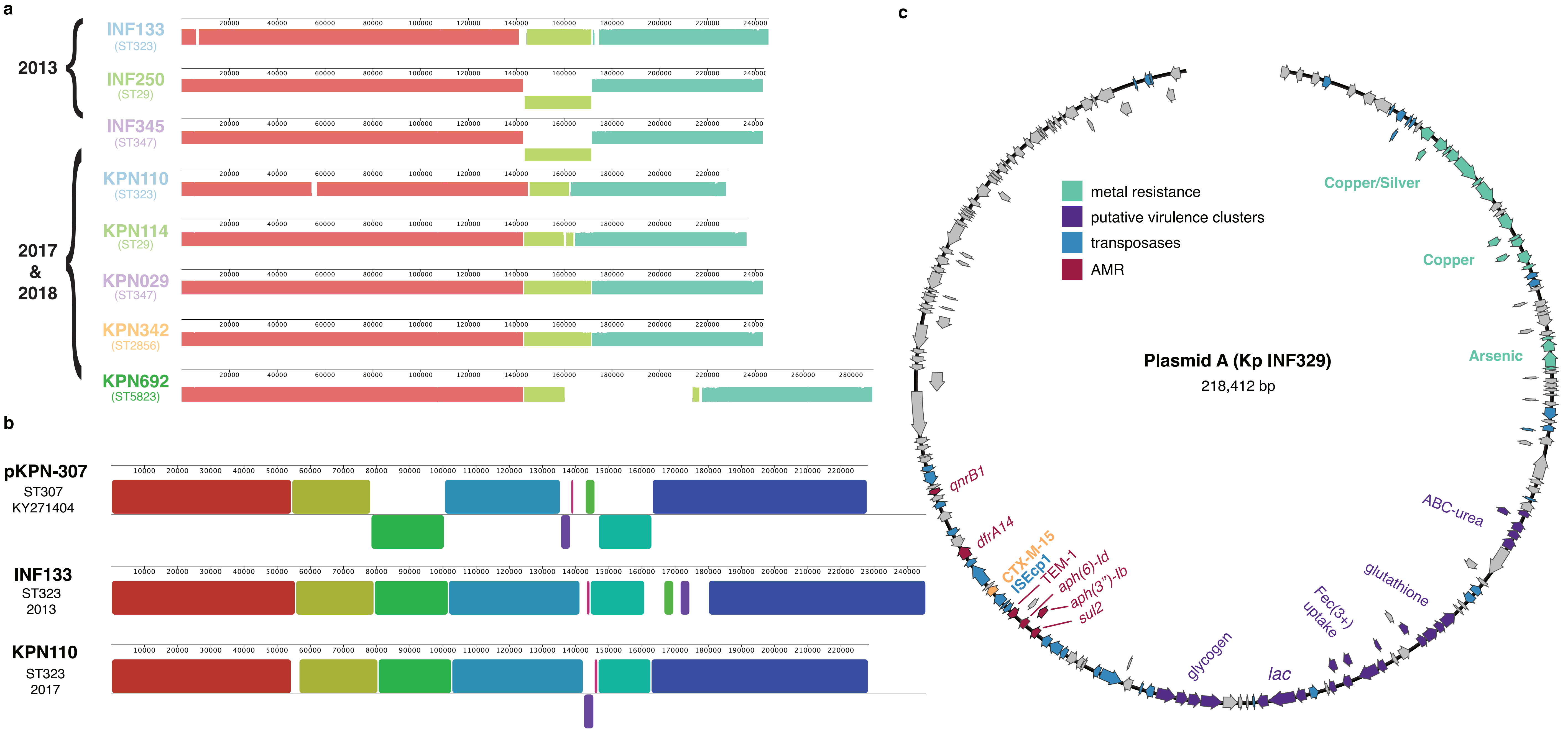

Supplementary Figure 4: Comparison of completed Plasmid A sequences. a, progressiveMauve alignment of completed Plasmid A sequences from one representative of ST323 (blue), ST29 (green) and *K. variicola* ST347 (purple) from 2013, and one representative of ST323, ST29, ST347, ST2856 and ST5823 from 2017 or 2018. **b,** progressiveMauve alignment of the $bla_{CTX\text{-}M\text{-}15}$ plasmid found in *K. pneumoniae* ST307 (accession KY271404) with Plasmid A from ST323 (in both 2013 and 2017). **c,** Gene map of Plasmid A in INF329 (ST323). Genes are represented by arrows and coloured by function as per legend. Putative virulence clusters are the same as those defined by Villa et al.

a

patient	ID	species	ST	infection	Carbapenemase plasmid	ESBL plasmid
AH0269	MSB1_3B	*E. coli*	ST38	carriage	Plasmid M (*bla* OXA-48)	Plasmid K (*bla* CTX-M-27)
AH0269	INF298	*K. pneumoniae*	ST231	infection	Plasmid M (*bla* OXA-48)	Plasmid B (*bla* CTX-M-15)
AH0269	KSB1_5H	*K. pneumoniae*	ST231	carriage	Plasmid M (*bla* OXA-48)	Plasmid B (*bla* CTX-M-15)
AH0269	MSB1_8A	*K. pneumoniae*	ST231	carriage	Plasmid M (*bla* OXA-48)	Plasmid B (*bla* CTX-M-15)
KC0227	INF281	*K. pneumoniae*	ST231	infection	Plasmid M (*bla* OXA-48)	Plasmid B (*bla* CTX-M-15)
KC0227	INF305	*K. pneumoniae*	ST231	infection	Plasmid M (*bla* OXA-48)	Plasmid B (*bla* CTX-M-15)
KC0242	MINF_6B	*K. pneumoniae*	ST231	infection	Plasmid M (*bla* OXA-48)	Plasmid B (*bla* CTX-M-15)
KC0245	INF299	*K. pneumoniae*	ST231	infection	Plasmid M (*bla* OXA-48)	Plasmid B (*bla* CTX-M-15)
KC0245	INF310	*K. pneumoniae*	ST231	infection	Plasmid M (*bla* OXA-48)	Plasmid B (*bla* CTX-M-15)
AH0095	INF157	*K. pneumoniae*	ST340	infection	Plasmid N (*bla* IMP-4)	Plasmid D (*bla* CTX-M-15)
AH0095	INF158	*K. pneumoniae*	ST340	infection	Plasmid N (*bla* IMP-4)	Plasmid D (*bla* CTX-M-15)
AH0095	KSB1_5D	*K. pneumoniae*	ST340	carriage	Plasmid N (*bla* IMP-4)	Plasmid D (*bla* CTX-M-15)
KC0221	INF164	*K. pneumoniae*	ST340	infection	Plasmid N (*bla* IMP-4)	Plasmid D (*bla* CTX-M-15)

b

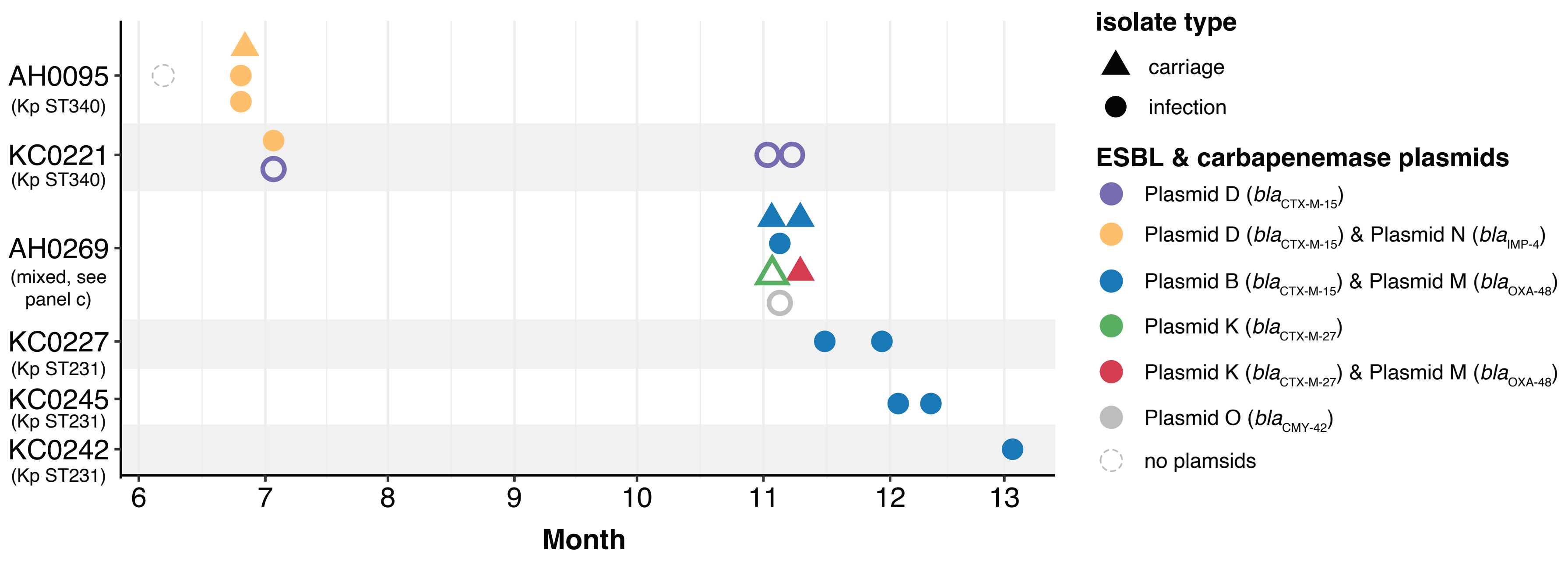

c

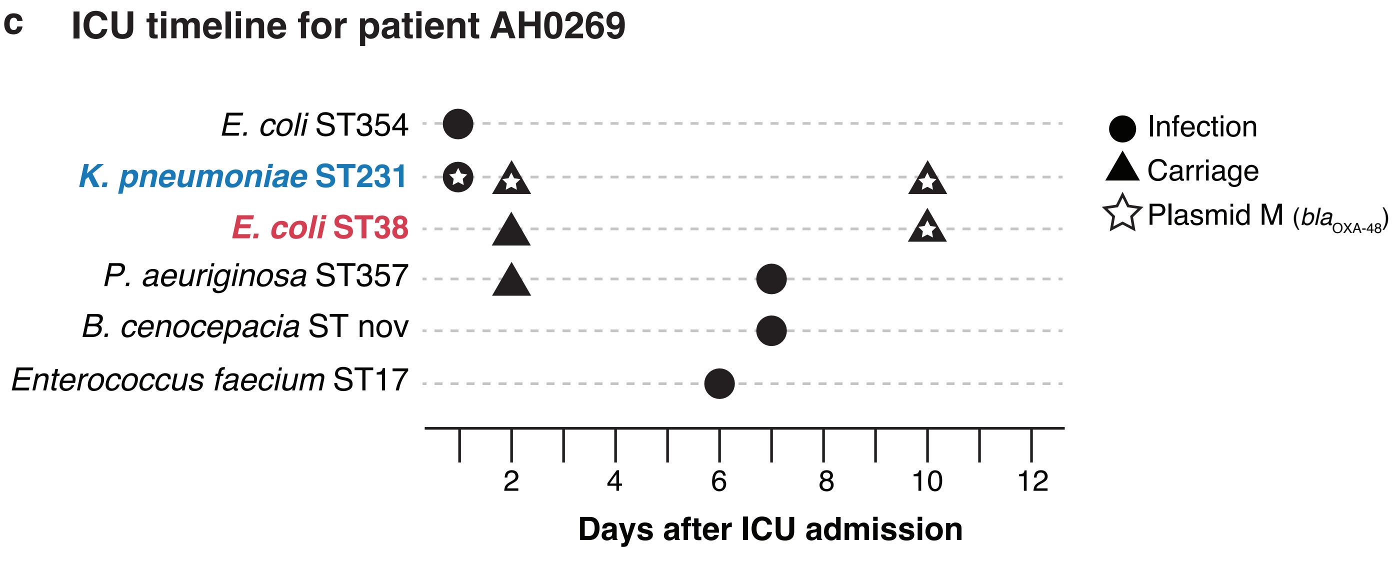

Supplementary Figure 5: Carbapenemase isolates and plasmids found in this study. a, Description of all carbapenemase isolates. **b,** Timeline of patients with carbapenemase isolates. Rows represent patient timelines, and each isolate is represented by a circle (infection) or triangle (carriage). Shapes are coloured by plasmid status as per legend. Filled shapes indicate the isolate harbours both an ESBL and carbapenemase plasmid, unfilled indicates the isolate carries only an ESBL plasmid. Dashed, unfilled grey circle indicates a *K. pneumoniae* ST280 infection isolate with no ESBL or carbapenemase plasmids. The strain carrying the plasmids is indicated in brackets under the patient ID (Kp – *K. pneumoniae*). **c,** Timeline for ICU stay of patient AH0269. Each row indicates a different bacterial lineage colonising this patient, with circles (infection) and triangles (carriage) representing the sampled isolates. White stars indicate an isolate carrying Plasmid M encoding the carbapenemase bla_{OXA-48}.
